# Modeling the Impact on HIV Incidence of Combination Prevention Strategies among Men Who Have Sex with Men in Beijing, China

**DOI:** 10.1371/journal.pone.0090985

**Published:** 2014-03-13

**Authors:** Jie Lou, Meridith Blevins, Yuhua Ruan, Sten H. Vermund, Sanyi Tang, Glenn F. Webb, Bryan E. Shepherd, Xiong He, Hongyan Lu, Yiming Shao, Han-Zhu Qian

**Affiliations:** 1 Department of Mathematics, Shanghai University, Shanghai, China; 2 Vanderbilt Institute for Global Health, Vanderbilt University, Nashville, Tennessee, United States of America; 3 Department of Biostatistics, Vanderbilt University, Nashville, Tennessee, United States of America; 4 State Key Laboratory for Infectious Disease Prevention and Control, National Center for AIDS/STD Control and Prevention, Chinese Center for Disease Control and Prevention, Collaborative Innovation Center for Diagnosis and Treatment of Infectious Diseases, Beijing, China; 5 Department of Pediatrics, Vanderbilt University, Nashville, Tennessee, United States of America; 6 College of Mathematics and Information Science, Shaanxi Normal University, Xi'an, Shaanxi Province, China; 7 Department of Mathematics, Vanderbilt University, Nashville, Tennessee, United States of America; 8 Institute for AIDS/STD Prevention & Control, Beijing Municipal Center for Disease Control and Prevention, Beijing, China; 9 Division of Epidemiology, Department of Medicine, Vanderbilt University, Nashville, Tennessee, United States of America; Old Dominion University, United States of America

## Abstract

**Objective:**

To project the HIV/AIDS epidemics among men who have sex with men (MSM) under different combinations of HIV testing and linkage to care (TLC) interventions including antiretroviral therapy (ART) in Beijing, China.

**Design:**

Mathematical modeling.

**Methods:**

Using a mathematical model to fit prevalence estimates from 2000–2010, we projected trends in HIV prevalence and incidence during 2011–2020 under five scenarios: (S1) current intervention levels by averaging 2000–2010 coverage; (S2) increased ART coverage with current TLC; (S3) increased TLC/ART coverage; (S4) increased condom use; and (S5) increased TLC/ART plus increased condom use.

**Results:**

The basic reproduction number based upon the current level of interventions is significantly higher than 1 (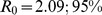 confidence interval (CI), 1.83–2.35), suggesting that the HIV epidemic will continue to increase to 2020. Compared to the 2010 prevalence of 7.8%, the projected HIV prevalence in 2020 for the five prevention scenarios will be: (S1) Current coverage: 21.4% (95% CI, 9.9–31.7%); (S2) Increased ART: 19.9% (95% CI, 9.9–28.4%); (S3) Increased TLC/ART: 14.5% (95% CI, 7.0–23.8%); (S4) Increased condom use: 13.0% (95% CI, 9.8–28.4%); and (S5) Increased TLC/ART and condom use: 8.7% (95% CI, 5.4–11.5%). HIV epidemic will continue to rise (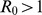) for S1–S4 even with hyperbolic coverage in the sensitivity analysis, and is expected to decline (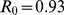) for S5.

**Conclusion:**

Our transmission model suggests that Beijing MSM will have a rapidly rising HIV epidemic. Even enhanced levels of TLC/ART will not interrupt epidemic expansion, despite optimistic assumptions for coverage. Promoting condom use is a crucial component of combination interventions.

## Introduction

More than 30 years have passed since the first HIV case was reported among men who have sex with men (MSM) [1]. MSM contribute to the majority of new cases in the Americas, Western Europe, Oceania, and much of Asia [2]. Of the 780,000 people living with HIV/AIDS in China, 17.4% were infected through homosexual contacts in 2011, rising from 11.0% in 2007 and 7.3% in 2005 [3]. A recent survey among 47,231 MSM from 61 Chinese cities showed an overall prevalence of 4.9% [4]. The unrelenting HIV burden among MSM suggests that HIV intervention remains an urgent priority. One promising concept is the expansion of HIV testing and linkage to care (TLC) including antiretroviral therapy (ART), so that a much higher proportion of infected men know their HIV status and undergo ART, thereby reducing their infectiousness to others [5], [6], [7], [8], [9].

China has implemented several aggressive public health programs in the past decade, including routine HIV testing and risk reduction interventions for high risk populations and free ART for HIV-infected treatment-eligible individuals [10]. These programs curtailed HIV transmission through unhygienic plasma collection and injecting drug use, and reduced mortality among HIV-infected patients [11], [12], [13]; however, no programs have reversed the rising HIV epidemic among Chinese MSM [14], [15]. A recently published mathematic model estimated that a four-fold increase in testing rates may prevent 42,000 new HIV infections among all at-risk groups in China over 5 years [16]. A few mathematical models evaluated independent or joint impacts of HIV testing, risk reduction intervention and ART among MSM [17], [18], [19], [20], [21], [22], [23], [24], but these studies were mainly from the Americas, Western Europe, and Australia, where the HIV epidemics have been long established among MSM, and none from regions with a rapidly rising HIV epidemic among MSM. ART may not have the same protection against HIV transmission in homosexual contacts as in heterosexual contacts due to biological and behavioral differences in infectiousness/susceptibility or differences in co-factor frequency [25]. Furthermore, the effectiveness of combined TLC intervention packages may be contingent on the epidemiological context [26].

In this study, we modeled the dynamics of the HIV/AIDS epidemic among MSM in China's capital city, Beijing, which is representative of most Chinese urban areas [4]. We simulated HIV prevalence from 2000–2010 based on the coverage and intensity of existing public health programs including uptake of HIV testing, linkage to HIV care (e.g. risk reduction), ART and condom use. Further, we made projections for the HIV epidemic beyond 2010 under various intervention scenarios. We then examined the robustness of model predictions based on different underlying assumptions in sensitivity analyses.

## Methods

We used a compartmental ordinary differential equations model for simulating and projecting the HIV epidemic among MSM in Beijing. Instead of the bilinear incidence [27], [28], [29] and the standard incidence models [5], [8], [9], we used *Bernoulli processes* to describe the probability of HIV transmission [30]. *Bernoulli processes* can more accurately describe the HIV transmission network among MSM by giving consideration for multiple sexual partners, type of sexual partner (i.e., regular or casual), differential condom use, and probability of transmission by preferred anal sex position (i.e., receptive or insertive). Substance abuse is rare among MSM in Beijing; therefore, injecting drug use is unlikely to contribute substantially to HIV transmission [31],[32],[33]. Additionally, some Chinese MSM may have female sexual partners, but these female partners generally do not have risky behavior and are unlikely to transmit disease to male partners [34]. Therefore, we only considered HIV transmission as a result of homosexual contact; we implicitly assumed there is no other route of transmission [31], [33], [35]. We also assumed that the age of active homosexual intercourse ranges from 18–60 years among Chinese MSM [36].

### Model structure

Receipt of HIV testing and risk reduction services may change the risk of HIV transmission among MSM. Thus, we divided the study population into three mutually exclusive sub-populations: those who are not testing for HIV (**N**on-testing), those who are testing but not reducing their risk of infection/transmission (**T**esting), and those who are testing and reducing their risk of infection/transmission (**R**isk reduction). The population was further compartmentalized by infection status and disease status (or eligibility for ART treatment). According to Chinese national ART guidelines [37], patients with CD4+ T cell count <350 cells/µL are eligible for ART treatment.

**Figure 1:**
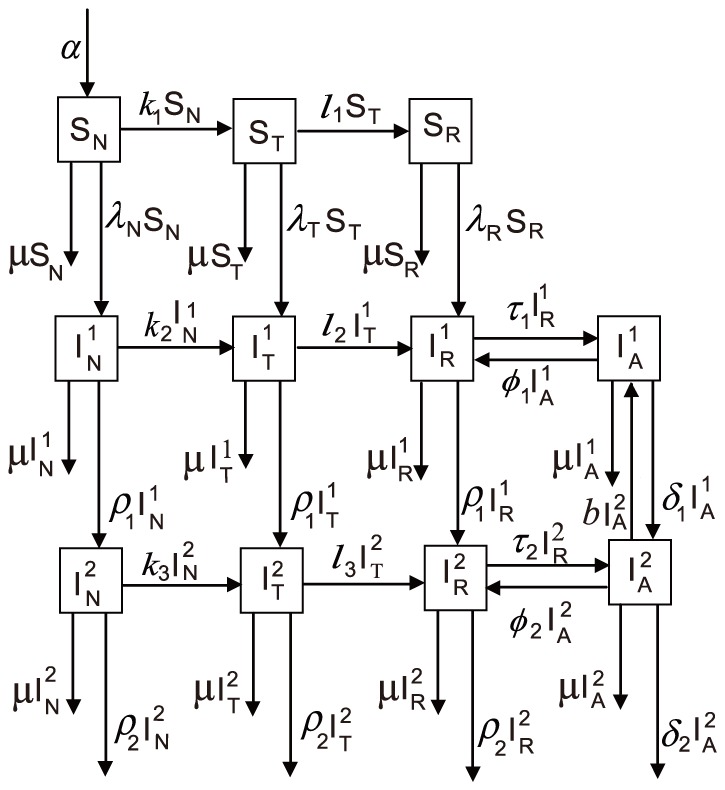
Schematic diagram of HIV combination prevention intervention model in the presence of ART. The top row of compartments show uninfected MSM who are **S**usceptible to HIV infection. They are divided into **N**on-testers, **T**esters, and testers who received **R**isk reduction and linkage-to-care interventions. The bottom two rows representing two stages of disease contain an additional compartment—**I**nfected individuals. Stage **1** is CD_4_+ cell count <350/µL while stage **2** is CD_4_+ ≥350/µL. The last column of compartments shows HIV-infected individuals who have initiated **A**ntiretroviral therapy. 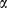 is the recruitment rate per year into the MSM population in Beijing. They are local HIV- MSM who turn 18 years old, or those from other areas who immigrate to Beijing. Horizontal movement is parameterized by rates of testing (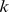), rates of linkage to care (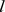), rates of ART initiation (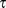), and rates of ART failure or dropout (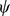). Movement from Susceptible to Infected (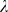) is the HIV transmission rate. It is estimated from multiple parameters including condom use, insertive/receptive intercourse, regular/casual partner encounter, CD4 count, and reductions in infectivity due to TLC or ART. The natural removal rate (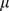) represents death, being older than 60 years, or migration out of Beijing. The rate of disease progression is different for HIV-infected individuals on ART (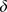) or not on ART (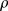).

Figure 1 shows the schematic diagram of our compartmental model structure. In this model we assumed that uninfected MSM enter the model as susceptible (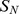) (these MSM could be local Beijing residents who turn 18 years old, or those who migrate from other areas to Beijing). Some who receive HIV testing may test negative (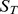), and then a proportion of those HIV-negative men are linked to care and receive risk reduction intervention provided by public health programs in Beijing (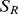). The subscripts 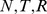 refer to the non-testing, testing, and risk reduction, respectively.

Upon infection, individuals move from susceptible into infected status, and the latter is further divided into two infection stages: CD4 count ≥350/µL (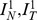 and 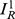) and CD4 count <350/µL (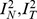 and 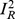). Flow is unidirectional from susceptible status to infection stage 1 and then 2, as without ART, an HIV-infected individual is unlikely to experience immunological recovery. A proportion of infected MSM (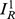 or 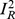) receive ART (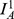 or 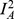); these compartments are bidirectional due to attrition or adherence to ART and decline or improvement of immunological status.

The key to quantifying HIV incidence among MSM in Beijing is specifying the time-dependent HIV transmission rate, 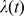. Multiplying the number of susceptible MSM by this transmission rate gives the frequency of new infections. Since MSM in Beijing often have multiple sexual partners, we classified them as regular partners (RP) and casual partners (CP), because they typically have different risk profiles [32], [35]. We also classified sexual behaviors of MSM as insertive or receptive anal intercourse (AI) with different HIV transmission probabilities per sex act [8]. We investigated the relationship between per-act and per-partner AI transmission probabilities for a given number of sex acts using the *Bernoulli process* assuming independence of risk for each sex act within a partnership [38], [39]. Parameter notation and descriptions are provided in Table 1; initial conditions and parameter values are described later.

**Table 1:** The notation, description, value and uncertainty range of parameters and their data sources for the mathematical model.

	Parameter	Description	Value	Uncertainty range	Source
Initial conditions	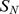	No. of non-tested susceptible MSM	102,271		
	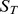	No. of tested susceptible MSM	128		
	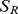	No. of risk reduction (RR) susceptible MSM	0		
	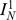	No. of non-tested MSM_+_ in infection stage 1	828		
	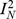	No. of non-tested MSM_+_ in infection stage 2	414		
	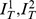	No. of tested MSM_+_	1		
	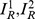	No. of RR MSM_+_	0		
	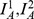	No. of MSM_+_ on antiretroviral therapy (ART)	0		
Progression	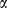	Recruitment rate into target population per year	5,012	2,073–6,633	Fit
	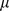	Natural removal rate of MSM_−_ per year	0.024	-	[8]
	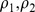	Progression rate of MSM_+_ without ART	0.18	-	[5]
	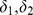	Progression rate of MSM_+_ with ART	0.089	-	[5]
Sex and condoms	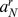	No. of anal intercourse (AI) for non-tested MSM_+_ with RP	36.5	32.2–40.1	[31], [32], [47]
	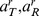	No. of AI for tested/RR HIV_−_ with RP	36.2	34.9–38.6	[31], [32], [47]
	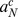	No. of AI for non-tested HIV_−_ with CP	18.2	11.3–21.7	[31], [32], [47]
	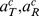	No. of AI for tested/RR HIV_−_ with CP	22.2	20.9–24.6	[31], [32], [47]
	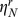	Condom use among non-tested MSM with RP	30.7%	28.0–36.7%	[31], [32], [34], [47]
	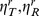	Condom use among tested/RR MSM with RP	31.6%	23.8–37.2%	[31], [32], [34], [47]
	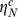	Condom use among non-tested MSM with CP	37.7%	34.5–42.3%	[31], [32], [34], [47]
	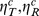	Condom use among tested/RR MSM with CP	41.4%	37.0–43.7%	[31], [32], [34], [47]
Transmission	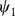	Relative infectiousness (RI) of tested MSM_+_ to non-tested MSM_+_	0.74	0–1	[31], [32]
	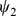	RI of RR MSM_+_ to non-tested MSM_+_	0.48	0–1	Fit
	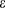	RI of MSM_+_ on ART to not on ART	0.4	0.01–.4	[48]
	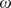	RI of MSM_+_ in infection stage 2 to stage 1	1.63	0.5–3	Fit
	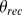	New infections due to unprotected receptive AI	0.016	0.008–0.028	Fit
	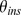	New infections due to unprotected insertive AI	0.0012	0.0006–0.0019	Fit
	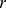	Sex acts of insertive AI	0.56	0.50–0.61	Fit
Intervention uptake	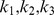	Testing rates among MSM_−_ or MSM_+_	0.09	0.05–0.20	[49]
	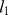	Linkage to risk reduction (RR) rate among MSM_−_	0.19	0.10–0.25	Fit
	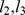	Linkage to risk reduction (RR) rates among MSM_+_	0.33	0.25–0.5	[49]
	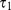	ART initiation rate among linked MSM_+_ in their infection stage 1	0.018	0.005–0.036	[49]
	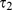	ART initiation rate among linked MSM_+_ in their infection stage 2	0.53	0.44–0.62	[49]
	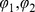	ART dropout rate	0.024	-	[49]
	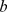	Immunological recovery on ART	0.50	0–1	Fit

**Note**: MSM_+_: HIV-infected MSM; MSM_+_: HIV-uninfected MSM; RP: regular sexual partner; CP: casual sexual partner.

Using the *Bernoulli process* model of HIV transmission during a given number of sex acts, the non-infection probability by infected regular sexual partners through receptive AI 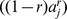 and insertive AI (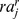) under condom-use rate 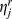 can be described as:
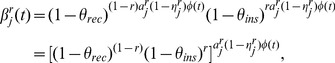
(1)where *r* is the proportion of sex acts which are insertive AI and *θ* is the single act transmission probability, and 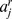 and 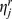 are the mean number of sex acts and condom use rates with superscripts *r* (above) or *c* (not shown) denoting regular or casual sex partnerships, respectively.

The HIV transmission rate is defined as:
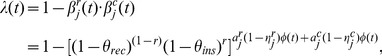
(2)where 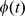 is the effective contact rate for all MSM *N* at time *t* with
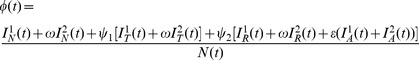
(3)


The definitions for parameters in the equation (3) are described in Table 1.

From this model, we calculated the basic reproduction number (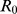) [40]. 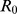 is the number of secondary cases produced by a typical HIV-infected MSM during his entire period of infectiousness in a demographically steady susceptible population. Calculating 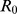 is critical to determine whether HIV will increase, stabilize, or decline among the MSM population in Beijing.

### Data source and estimation

To simulate the HIV prevalence rates during 2000–2010 among MSM in Beijing, we used data from local HIV surveillance systems and published studies [31], [32], [33], [35], [41]. As these data were summary statistics, and did not include any identifiers that could link the data to individual subjects in the local HIV surveillance systems or participants from the published studies, consent was waived and the mathematical modeling protocol was approved by the institutional review boards of the National Center for AIDS/STD Control and Prevention of Chinese Center for Disease Control and Prevention and Vanderbilt University.

We employed a Metropolis-Hastings algorithm to carry out extensive Markov-chain Monte-Carlo simulations for estimating the mean values of some unknown parameters [42], including yearly recruitment rate (or number of new members into the pool of the study population each year), risk-reduction rate of healthy MSM (during 2000–2010), relative infectiousness of risk-reduction to non-testing subgroups, the per-act probability of unprotected insertive or receptive anal intercourse resulting in an infection, and the relative infectiousness ratio of HIV positive MSM in the infection stages 2 to 1 (see Figure 1). The algorithm ran for 1,000,000 iterations, and we adapted the proposal distribution after 500,000 iterations using Geweke's method to assess convergence [42].

#### Initial conditions

The earliest HIV prevalence data among MSM in Beijing were available for 2000. The observed HIV prevalence rates among MSM in Beijing during 2000–2010 were: 1.2% in 2000 [41], 3.2% in 2005 [32], 4.8% in 2006 [32], 5.1% in 2007 [32], 6.5% in 2008 [31], [33], 6.8% in 2009 [35], and 7.8% in 2010 [43]. In order to set the initial conditions, we had to estimate the target population of MSM living in Beijing in 2000. According the national census in 2000, there were 14 million of people living in Beijing. We assumed half of them were male and 3% of males in Beijing in 2000 were MSM, of which half lived in the city. The testing rate in 2010 was very low (assumed 0.125%). Also, we assumed there was no risk reduction intervention in 2000, as the burgeoning HIV epidemic in MSM did not receive attention from Chinese public health programs at that time [44]. For these non-testing HIV-positive MSM, we assumed one-third were in infection stage 1 (CD4 count≥350/µL) and two-thirds were in the infection stage 2 (CD4 count <350/µL). Also, we assumed that there was no ART available for MSM in 2000. The resulting parameter values for the initial conditions are shown in Table 1.

#### Progression

With an 18 year average age of sexual debut, we considered a natural removal rate per year which corresponds to an average of 42 years of sexual activity and non-AIDS mortality (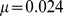). The recruitment rate per year into HIV-negative MSM population was estimated to range from 2–6.4% of the total population [8] giving the recruitment rate (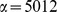) (Table 1). The progression duration from HIV infection to AIDS is 10–12 years in the absence of treatment and may increase by additional 8–12 years with ART [5], [45], [46]. We used estimated disease progression rates being an average duration of 5.6 years without ART (therefore, 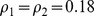), and 11.2 years with ART (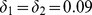) [5]. We also performed sensitivity analyses for a range of disease progression rates.

#### Sex and condom use

We estimated condom use rate and frequency of AI based on three cohort studies among MSM in Beijing [31], [32], [47] and one meta-analysis among Chinese MSM [34]. Table 1 shows condom use rates for insertive and receptive AI (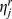 and 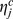) ranging from 30.7%–41.4% [34] and the average frequency of AI with regular and casual sexual partners (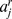 and 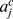) ranging from 18.2–36.6 [31], [32], [47].

#### Transmission

Relative infectiousness of HIV-infected MSM (MSM_+_) who receive HIV testing versus non-testing was defined as the ratio of high risk behavior of testing MSM_+_ to that of non-testing MSM_+_. The rationale is that HIV testing (and counseling) may lead to change of risky behaviors, such as reduction in sexual encounters or increasing condom use, or both. Based on risky behaviors among MSM_+_ from three cohort studies [31], [32], [42], we calculated the relative infectiousness ratio (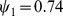; 13.8/18.6) (Table 1). We assumed that non-testing MSM did not have different risk behaviors by HIV status (as they are unaware of their status) and simulated the effect of risk reduction intervention to non-testing MSM (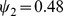). The conservative estimate for the relative infectiousness among MSM_+_ who are on ART versus non-ART (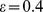) was based on evidence that ART can reduce HIV transmission by 60% [48].

The per-act risk of HIV transmission through unprotected receptive AI ranged from 0.008–0.028, while the risk through insertive AI ranged from 0.0006–0.0019 [8]. The estimated proportion of practicing receptive anal sex among MSM ranged from 0.4–0.5 while the proportion of practicing insertive sex ranged from 0.5–0.6 [8], and we estimated the proportion of insertive versus receptive sex acts as 0.56 (Table 1).

#### Intervention uptake

The average testing rate for MSM during 2001–2010 was estimated to be 9% [49]. The linkage rates for HIV-positive MSM ranged from 17% to 85% with a mean rate of 33% [49]. The ART initiation rates were estimated to be 1.8% and 53% among MSM whose CD4 counts are ≥350/µL and <350/µL, respectively [49].

### Hypothetical scenarios

We used the model to project HIV prevalence and incidence trends during 2011–2020 under five different scenarios:

1. Maintaining the coverage of interventions as they were (i.e., average coverage during 2000–2010): ART coverage (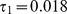 and 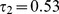), condom use (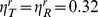 and 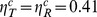), HIV testing (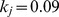), and linkage to care (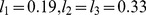) [31], [32], [34], [47], [49].
2. Increased ART coverage only (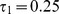 and 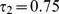).
3. Increased TLC/ART, but no increased condom use (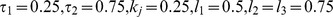).
4. Increased condom use only (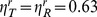 and 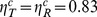).
5. Increased TLC/ART (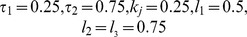) and condom use (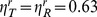 and 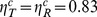).

In S4–S5, we assumed that condom use doubled since 2000.

### Sensitivity analysis

Finally, we performed sensitivity analyses to assess the robustness of the model results. First, by perturbing individual parameters, we investigated their influence on estimates of 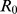. Second, we estimated uncertainty ranges for model parameters based on literature reviews (Table 1) and randomly sampled 20,000 parameter sets over these uncertainty ranges using a Latin Hypercube Sampling design [50], [51] to derive a frequency distribution for *R*_0_. Analyses were performed using Matlab computer software (MATLAB 7.7, The MathWorks Inc., Natick, MA, 2008); analysis scripts and supplemental materials are available online (http://biostat.mc.vanderbilt.edu/ArchivedAnalyses).

## Results

**Figure 2:**
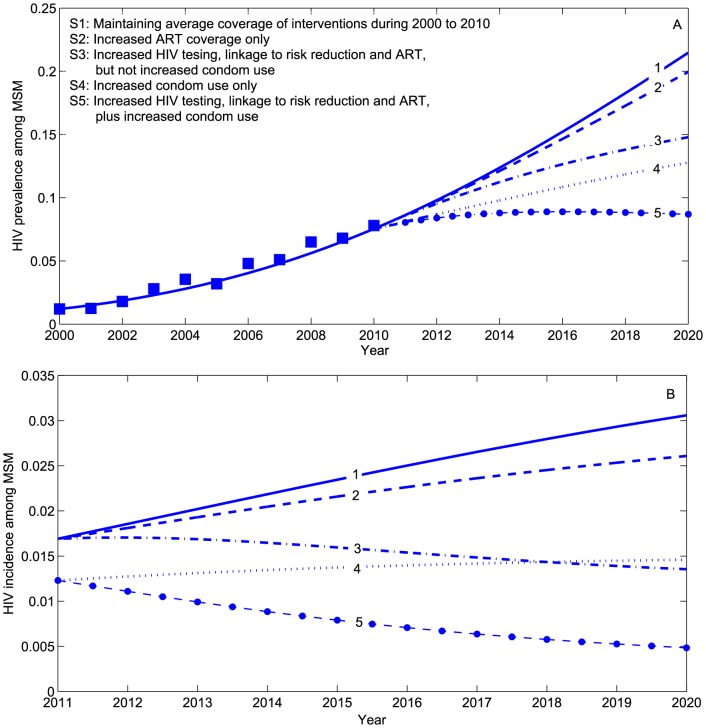
Predicted effects on HIV prevalence and incidence among Chinese MSM in five scenarios of HIV combination interventions. (A) effect on HIV prevalence; (B) effect on HIV incidence.

Using parameter values summarized in Table 1, our model produces a best-fit curve to historical HIV prevalence rates among MSM in Beijing during 2000–2010 (Figure 2A). The basic reproduction number in 2010 is 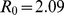 (95% confidence interval (CI), 1.83–2.35). Because 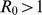, if the epidemic assumptions remain unchanged from 2010, the HIV prevalence is expected to substantially increase among MSM in Beijing. Using this best-fit model of HIV transmission during 2000–2010, we project HIV prevalence for the next ten years (until 2020) under five different scenarios. The HIV prevalence is 7.8% in 2010. If there is no change in the coverage of interventions or maintaining the average level during 2000–2010 (Figure 2, line 1), then the HIV prevalence is estimated to increase to 21.4% (95%CI, 9.9–31.7%) (Figure 2A, line 1), with an incidence rate of 3.1% (95%CI, 1.0–5.1%) by 2020 (Figure 2B, line 1). By increasing the coverage of ART among MSM_+_ who are linked to risk reduction (Figure 2, line 2), we expect a modest improvement in lowering HIV prevalence (19.9%; 95%CI, 9.9–28.4%) and incidence (2.6%; 95%CI, 0.8–4.6%). If TLC and ART coverage increases (Figure 2, line 3), the projected HIV prevalence and incidence will be 14.5% (95%CI, 7.0–23.8%) and 1.4% (95%CI, 0.2–3.3%). If MSM increase condom use only (Figure 2, line 4), then HIV prevalence and incidence will be 13.0% (95%CI, 9.8–28.4%) and 1.5% (95%CI, 0.8–4.6%), respectively. Bundling increased TLC/ART with increased condom use (Figure 2, line 5) may result in an HIV prevalence of 8.7% (95%CI, 5.4–11.5%) and an incidence of 0.5% (95%CI, 0.1–0.9%) by 2020. It is important to note that only this bundled HIV intervention strategy is likely to have an 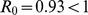 and eventually result in HIV eradication in this population.

**Figure 3:**
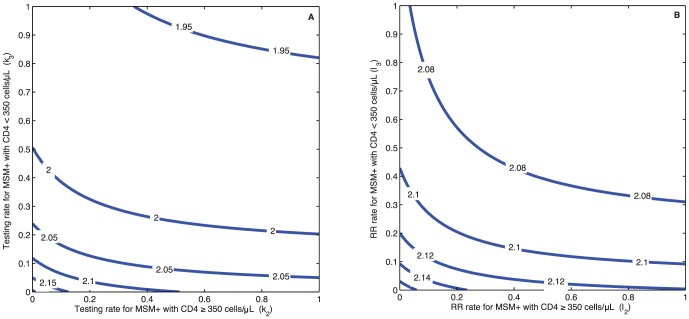
Contour plots: the effects on 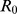 under different coverage rates of HIV testing and risk reduction interventions among HIV-infected Chinese MSM while condom use remains at the average level of years 2000–2010. (A) Under the different coverage rates of HIV testing intervention; (B) Under the different coverage rates of HIV risk reduction (RR) intervention.

We performed simple sensitivity analyses for intervention and transmission parameters. First, we analyzed the influence of the six intervention parameters (testing and linkage to risk reduction) on the prevalence during 2000–2020 (Table S1). The parameters related to HIV testing (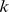) have the greatest influence on the estimates of 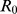. In particular, the partial rank correlation coefficient (PRCC) values range from −0.98 to −0.59, indicating that 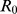 decreases for higher testing rates. Similarly, the parameters related to HIV linkage to risk reduction (*l*) also impact 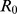. PRCC ranged from −0.44 to −0.14, indicating that 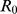 decreases for a higher linkage rate. Figure 3 shows two contour plots of 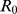 under different HIV testing or linkage to risk reduction among MSM_+_ while condom use remains at the average level during 2000–2010. Even if all MSM_+_ are tested (testing rate = 100%) or are linked to risk reduction (RR rate = 100%) without increasing condom use, the reproduction number remains above the epidemic threshold (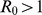).

Second, we analyzed the influence of the relative infectiousness of MSM_+_ on ART versus non-ART (varying 0.01–0.4) and that of MSM_+_ with CD4 counts <350/µL versus ≥350/µL (varying 0.5–5.0). In all perturbations for relative infectiousness, 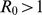 endures.

Third, we found that 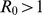 is sustained if MSM increase condom use but the coverage of TLC interventions remains at the average level during 2000–2010.

Fourth, we varied the average duration of disease progression without ART from 3.7–11.1 years and with ART from 7.4–22.2 years. The median 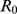 is 2.11 and consistently greater than 1.

In extensive sensitivity analyses, 20,000 parameter sets were randomly sampled from corresponding uncertainty ranges (Table S2). The distribution of 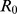 is skewed to the right with mean 1.93 (standard deviation: 0.82) and median 1.82 (interquartile range: 1.36–2.42). A small percentage (11.4%) of estimates falls below the epidemic threshold (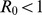). Relative infectiousness of RR to non-testing MSM_+_, transmission probability of unprotected receptive AI, and relative infectiousness of MSM_+_ on ART to non-ART are the three most influential parameters with PRCC equaling to 0.95, 0.90 and 0.71, respectively.

## Discussion

Combination testing and treatment intervention packages have been considered as a promising strategy for HIV prevention among MSM in recent years [52]. We explored the effects of TLC/ART, along with condom use, for the emerging HIV epidemic among Chinese MSM [4], [53]. Our projections predicted that with the current level of services HIV prevalence among MSM in Beijing will almost triple from 7.8% in 2010 to 21.4% by 2020. Expansion of ART coverage in the current continuum of care among MSM_+_ would only lead to a moderate reduction of this rising trend, though enhanced TLC interventions plus increased ART coverage will further reduce incident HIV infections. Only with a combination of increased condom use and a higher TLC/ART coverage might HIV epidemic be expected to decline. Hence, promoting safer sex education and risk reduction counseling service to increase condom use is crucial for HIV prevention among Chinese MSM, even in the context of expanded TLC and ART.

As observational studies and the HPTN 052 randomized controlled trial have demonstrated the effectiveness of ART for reducing heterosexual HIV transmission [54], [55], [56], [57], [58], treatment as prevention has been advocated as a strategy for HIV prevention among MSM [59]. However, the impact may vary by epidemic scenarios [21]. In settings with a long-standing epidemic like San Francisco, TLC with ART expansion may reduce the HIV epidemic [52]. Our model is less optimistic that ART interventions alone will succeed in substantial prevention reduction. The HIV epidemic among Chinese MSM is relatively recent [44], and the majority of cases may still be in earlier stages of disease, and therefore have not received ART [13]. We use local public health data such as ART coverage rates for estimating the parameters in the mathematical models; therefore our modeling results likely reflect what is actually happening in Beijing. A comparable example is that in the HPTN 052 trial earlier ART treatment reduced heterosexual transmission by 96% [7], while among 38,862 Chinese serodiscordant couples the rate of transmission was only reduced by 26% in the first year of treatment and the reduction lost statistical significance in subsequent years [60]. The former result comes from a controlled trial setting while the later one is from a real-world public health setting. Our model projections are consistent with the study in the United Kingdom where increased condom use was more likely to drive down HIV incidence than high ART coverage [20].

Increasing coverage of HIV testing may lead MSM_+_ in Beijing to reduce risky behaviors, notably the use of condoms; partner reduction interventions and serosorting (i.e., MSM of the same HIV status) interventions could conceivably have a similar benefit, but we did not model these factors. Based on our model, HIV prevalence will continue to increase, as fewer MSM_+_ die and fall out of transmission networks. The impact of HIV testing is largely dependent on how those infected MSM with known status reduce their risk behaviors, particularly practicing anal sex without condom use. Given that only a fraction of HIV-infected men know their status, are eligible for ART, and successfully negotiate the system to adhere to ART, increased condom use is a crucial element in the combined interventions for reducing the epidemic.

The limitation of our mathematical model is a simplification of the real world epidemic. Like other models, we balance parsimony and reality. For example, we assumed that all linked MSM_+_ receive risk reduction. This is consistent with Chinese HIV testing protocol that all MSM_+_ are expected to receive risk reduction counseling. However, in reality, the quality of counseling may vary by clinic and client. Because some counseling may not lead to behavioral changes, we considered condom use as an independent factor in our model. Although model parameters are carefully specified using the available literature, they may not reflect the current realities among Beijing MSM. For example for scenario 3, we assumed increasing HIV testing from 9% to 25%; increasing the linkage rate to risk reduction education from 19% to 50% among HIV-uninfected MSM and from 33% to 75% among HIV-infected MSM; increasing ART coverage from 1.8% to 25% among HIV-infected MSM whose CD4+ cell count ≥350/µL and from 53% to 75% among those with CD4 count <350/µL. The magnitude of these assumed increases in coverage are based on our best estimations of how well the public health system could do, in the view of local HIV/AIDS experts (including authors YR, XH, HL and YS as well as non-author experts from local centers for disease control). However, we performed extensive sensitivity analyses by varying input parameters and found that our findings are robust to reasonable perturbations.

No scenario can result in a substantial decline in HIV epidemic by 2020 among Chinese MSM, except the bundled package of transmission prevention strategies including increased TLC/ART and condom use. Simulations show that TLC alone has some impact for HIV reduction, but far less than increasing condom use. Our model suggests that increasing condom use must be a component of combination intervention packages to achieve significant reduction in HIV incidence.

## Supporting Information

Table S1 Partial Rank Correlation Coefficients for *R*_0_.
(DOCX)

Table S2 Sensitivity analysis of reproduction number *R*_0_.
(DOC)
