# Supplementary material for: Modeling the Impact on HIV Incidence of Combination Prevention Strategies among Men Who Have Sex with Men in Beijing, China
Source: PLoS One. 2014 Mar 13;9(3):e90985. doi: 10.1371/journal.pone.0090985 (PMC3953201; doi:10.1371/journal.pone.0090985)
Supplement: Table S1 — Partial Rank Correlation Coefficients for R 0. (DOCX) [file pone.0090985.s001.docx]

**Table S1: Partial Rank Correlation Coefficients for *$R_{0}$***

| Parameter | Partial Rank Correlation Coefficient | p-value | Importance |
| --- | --- | --- | --- |
| *$k_{1}$* | -0.98 | <0.001 | *$k_{1}$* |
| *$k_{2}$* | -0.87 | <0.001 | *$k_{2}$* |
| *$k_{3}$* | -0.59 | <0.001 | *$k_{3}$* |
| *$l_{1}$* | -0.44 | <0.001 | *$l_{1}$* |
| *$l_{2}$* | -0.33 | <0.001 | *$l_{2}$* |
| *$l_{3}$* | -0.14 | <0.001 | *$l_{3}$* |
