# Supplementary material for: Modeling the Impact on HIV Incidence of Combination Prevention Strategies among Men Who Have Sex with Men in Beijing, China
Source: PLoS One. 2014 Mar 13;9(3):e90985. doi: 10.1371/journal.pone.0090985 (PMC3953201; doi:10.1371/journal.pone.0090985)
Supplement: Table S2 — Sensitivity analysis of reproduction number R 0. (DOC) [file pone.0090985.s002.doc]

**Table S2: Sensitivity analysis of reproduction number** $R_{0}$

| Parameter | Correlation  Coeﬃcient | p-value | Importance |
| --- | --- | --- | --- |
| $k_{1}$_1_ | -0.22 | <0.001 | $\psi_{2}$ |
| $k_{2}$_2_ | -0.093 | <0.001 | $\theta_{rec}$ |
| $k_{3}$_3_ | -0.042 | <0.001 | $\varepsilon$ |
| $l_{1}$_1_ | -0.026 | <0.001 | $\omega$ |
| $l_{2}$_2_ | -0.015 | 0.04 | $b$ |
| $l_{3}$ 3 | -0.004 | 0.60 | $r$ |
| $\varepsilon$ | 0.71 | <0.001 | $k_{1}$ |
| $\omega$ | 0.65 | <0.001 | $\psi_{1}$ |
| $\psi_{2}$ | 0.95 | <0.001 | $\theta_{ins}$ |
| $\eta_{N}^{r}$ | -0.044 | <0.001 | $\eta_{N}^{r}$ |
| $\eta_{N}^{c}$ | -0.032 | <0.001 | $a_{N}^{c}$ |
| $\eta_{T}^{r}$ | -0.023 | <0.001 | $k_{2}$ |
| $\eta_{T}^{c}$ | -0.009 | 0.20 | $\tau_{1}$ |
| $\eta_{R}^{r}$ | -0.15 | <0.001 | $a_{N}^{r}$ |
| $\eta_{R}^{c}$ | -0.049 | <0.001 | $a_{R}^{r}$ |
| $a_{N}^{r}$ | 0.082 | <0.001 | $a_{R}^{c}$ |
| $a_{N}^{c}$ | 0.10 | <0.001 | $\eta_{R}^{c}$ |
| $a_{T}^{r}$ | 0.010 | 0.14 | $\eta_{N}^{r}$ |
| $a_{T}^{c}$ | 0.016 | 0.02 | $k_{3}$ |
| $a_{R}^{r}$ | 0.069 | <0.001 | $\eta_{N}^{c}$ |
| $a_{R}^{c}$ | 0.058 | <0.001 | $l_{1}$ |
| $\tau_{1}$ | -0.083 | <0.001 | $\eta_{T}^{r}$ |
| $\tau_{2}$ | -0.016 | 0.02 | $\tau_{2}$ |
| $\psi_{1}$ | 0.20 | <0.001 | $a_{T}^{c}$ |
| $\theta_{ins}$ | 0.16 | <0.001 | $l_{2}$ |
| $\theta_{rec}$ | 0.90 | <0.001 | $a_{T}^{r}$ |
| $r$ | -0.36 | <0.001 | $\eta_{T}^{c}$ |
| $\alpha$ | 0.002 | 0.75 | $l_{3}$ |
| $b$ | 0.404 | <0.001 | $\alpha$ |
